# Supplementary material for: Long QT syndrome‐associated calmodulin variants disrupt the activity of the slowly activating delayed rectifier potassium channel
Source: J Physiol. 2023 Jul 10;601(17):3739–64. doi: 10.1113/JP284994 (PMC10952621; doi:10.1113/JP284994)
Supplement: Supplementary file 1 — Statistical Summary Document [file TJP-601-3739-s002.docx]

**Manuscript Title:** Long QT syndrome-associated calmodulin variants disrupt the activity of the slowly activating delayed rectifier potassium channel (IKs).

**Authors:** Liam McCormick, Kirsty Wadmore, Amy Milburn, Nitika Gupta, Rachael Morris, Marie Held, Ohm Prakash, Joseph Carr, Richard Barrett-Jolley, Caroline Dart, Nordine Helassa

**Animal model used, if applicable:** n/a

**Underlying hypothesis:** This investigation tests the hypothesis that calmodulin variants associated with long QT syndrome alter IKs activity.

**Definitions of ‘n’:**

Question 1-2: n = number of individual cells, from at least 3 independent experiments

Question 3-16: n = number of experimental replicates

**Statistical summary table:**

| Experimental question number* | Finding/ conclusion | Experimental location/ variable  e.g. muscle, neocortex or genotype | Mean value  (or other summary statistic) | SD | n val. | P** | Units | Data comparisons  e.g. WT vs KO | Statistical test | Figure/ table in which data are presented |
| --- | --- | --- | --- | --- | --- | --- | --- | --- | --- | --- |
| 1. Effect of LQTS-associated CaM variants on IKs at resting [Ca^2+^]? | Peak current densities (at +100 mV) reduced for all mutants | CaM-WT | 476.1 | 229.0 | 5 | - | pA/pF | WT vs mutants | Two-way ANOVA, Dunnett’s multiple comparisons tests | 2 |
|  |  | D95V | 220.5 | 122.7 | 11 | **<0.0001** |  |  |  |  |
|  |  | N97I | 347.0 | 68.2 | 8 | **0.0065** |  |  |  |  |
|  |  | D131H | 312.9 | 123.4 | 11 | **0.0001** |  |  |  |  |
|  | V_1/2_ activation increased for D95V and D131H variants | CaM-WT | 13.1 | 9.0 | 5 | - | mV | WT vs mutants | One-way ANOVA, Dunnett’s multiple comparisons tests | 2 |
|  |  | D95V | 34.4 | 13.3 | 11 | **0.0102** |  |  |  |  |
|  |  | N97I | 14.9 | 5.3 | 7 | 0.9878 |  |  |  |  |
|  |  | D131H | 41.0 | 16.1 | 11 | **0.0008** |  |  |  |  |
| 2. Effect of LQTS-associated CaM variants on IKs at high [Ca^2+^]? | Peak current densities (at +100 mV) reduced for all mutants | CaM-WT | 505.4 | 228.0 | 9 | - | pA/pF | WT vs mutants | Two-way ANOVA, Dunnett’s multiple comparisons tests | 3 |
|  |  | D95V | 341.1 | 132.2 | 12 | **0.0002** |  |  |  |  |
|  |  | N97I | 331.9 | 136.7 | 11 | **<0.0001** |  |  |  |  |
|  |  | D131H | 415.9 | 178.1 | 8 | 0.1118 |  |  |  |  |
|  | V_1/2_ activation increased for D95V and D131H variants | CaM-WT | 22.2 | 8.6 | 5 | - | mV | WT vs mutants | One-way ANOVA, Dunnett’s multiple comparisons tests | 3 |
|  |  | D95V | 31.6 | 9.3 | 12 | **0.0331** |  |  |  |  |
|  |  | N97I | 22.5 | 6.9 | 11 | 0.9996 |  |  |  |  |
|  |  | D131H | 32.1 | 7.4 | 8 | **0.0427** |  |  |  |  |
| 3. Effect of LQTS-associated CaM variants on Kv7.1 cell surface density? | No effect | CaM-WT | 35.0 | 2.6 | 5 | - | % | WT vs mutants | One-way ANOVA, Dunnett’s multiple comparisons tests | 4 |
|  |  | D95V | 30.8 | 5.8 | 5 | 0.3140 |  |  |  |  |
|  |  | N97I | 30.4 | 3.8 | 5 | 0.2502 |  |  |  |  |
|  |  | D131H | 35.4 | 4.3 | 5 | 0.9976 |  |  |  |  |
| 4. Effect of LQTS-associated mutations on CaM alpha-helices content (Ca^2+^-free)? | Alpha-helices content altered for N97I and D131H variants | CaM-WT | 35.9 | 0.4 | 5 | - | % | WT vs mutants | Two-way ANOVA, Dunnett’s multiple comparisons tests | 5 |
|  |  | D95V | 37.6 | 1.1 | 5 | 0.2817 |  |  |  |  |
|  |  | N97I | 31.5 | 4.2 | 5 | **0.0002** |  |  |  |  |
|  |  | D131H | 38.5 | 0.5 | 5 | **0.0386** |  |  |  |  |
| 5. Effect of LQTS-associated mutations on CaM alpha-helices content (Ca^2+^-bound)? | Alpha-helices content reduced for D131H variant | CaM-WT | 59.0 | 0.9 | 5 | - | % | WT vs mutants | Two-way ANOVA, Dunnett’s multiple comparisons tests | 5 |
|  |  | D95V | 58.0 | 0.4 | 5 | 0.0639 |  |  |  |  |
|  |  | N97I | 58.9 | 0.3 | 5 | 0.9751 |  |  |  |  |
|  |  | D131H | 41.5 | 0.6 | 5 | **<0.0001** |  |  |  |  |
| 6. Effect of LQTS-associated mutations on CaM protease degradation susceptibility (Ca^2+^-free)? | Protease degradation susceptibility (1 μg/ml trypsin) altered for all mutants | CaM-WT | 0.16 | 0.03 | 4 | - | fraction | WT vs mutants | Two-way ANOVA, Dunnett’s multiple comparisons tests | 7 |
|  |  | D95V | 0.05 | 0.03 | 4 | **<0.0001** |  |  |  |  |
|  |  | N97I | 0.05 | 0.02 | 4 | **<0.0001** |  |  |  |  |
|  |  | D131H | 0.27 | 0.07 | 4 | **<0.0001** |  |  |  |  |
| 7. Effect of LQTS-associated mutations on CaM protease degradation susceptibility (Ca^2+^-bound)? | Protease degradation susceptibility (10 μg/ml trypsin) increased for all mutants | CaM-WT | 0.59 | 0.02 | 3 | - | fraction | WT vs mutants | Two-way ANOVA, Dunnett’s multiple comparisons tests | 7 |
|  |  | D95V | 0.51 | 0.02 | 3 | **<0.0001** |  |  |  |  |
|  |  | N97I | 0.44 | 0.01 | 3 | **<0.0001** |  |  |  |  |
|  |  | D131H | 0.03 | 0.02 | 3 | **<0.0001** |  |  |  |  |
| 8. Effect of LQTS-associated mutations on CaM thermostability (Ca^2+^-free)? | No effect | CaM-WT | 41.79 | 0.38 | 3 | - | ^o^C | WT vs mutants | One-way ANOVA, Dunnett’s multiple comparisons tests | 7 |
|  |  | D95V | 42.57 | 0.26 | 3 | 0.0899 |  |  |  |  |
|  |  | N97I | 42.27 | 0.35 | 3 | 0.3446 |  |  |  |  |
|  |  | D131H | 41.08 | 0.35 | 3 | 0.1290 |  |  |  |  |
| 9. Effect of LQTS-associated mutations on CaM affinity for Kv7.1-Helix B (Ca^2+^-free)? | *K*_d_ increased for D95V and D131H variants | CaM-WT | 2.1 | 0.3 | 5 | - | μM | WT vs mutants | One-way ANOVA, Dunnett’s multiple comparisons tests | 9 |
|  |  | D95V | 3.9 | 0.3 | 6 | **<0.0001** |  |  |  |  |
|  |  | N97I | 2.4 | 0.5 | 5 | 0.6303 |  |  |  |  |
|  |  | D131H | 7.6 | 0.6 | 6 | **<0.0001** |  |  |  |  |
| 10. Effect of LQTS-associated mutations on CaM:Kv7.1-Helix B thermodynamic parameters, ΔG (Ca^2+^-free)? | ΔG increased for D95V and D131H variants | CaM-WT | -7.74 | 0.09 | 5 | - | kcal/mol | WT vs mutants | One-way ANOVA, Dunnett’s multiple comparisons tests | 9 |
|  |  | D95V | -7.39 | 0.05 | 6 | **<0.0001** |  |  |  |  |
|  |  | N97I | -7.67 | 0.11 | 5 | 0.3112 |  |  |  |  |
|  |  | D131H | -6.99 | 0.05 | 6 | **<0.0001** |  |  |  |  |
| 11. Effect of LQTS-associated mutations on CaM:Kv7.1-Helix B thermodynamic parameters, ΔH (Ca^2+^-free)? | ΔH reduced for all variants | CaM-WT | 7.63 | 0.23 | 5 | - | kcal/mol | WT vs mutants | One-way ANOVA, Dunnett’s multiple comparisons tests | 9 |
|  |  | D95V | 5.82 | 0.20 | 6 | **<0.0001** |  |  |  |  |
|  |  | N97I | 4.66 | 0.21 | 5 | **<0.0001** |  |  |  |  |
|  |  | D131H | 3.32 | 0.24 | 6 | **<0.0001** |  |  |  |  |
| 12. Effect of LQTS-associated mutations on CaM:Kv7.1-Helix B thermodynamic parameters, -TΔS (Ca^2+^-free)? | -TΔS increased for all variants | CaM-WT | -15.40 | 0.29 | 5 | - | kcal/mol | WT vs mutants | One-way ANOVA, Dunnett’s multiple comparisons tests | 9 |
|  |  | D95V | -13.18 | 0.19 | 6 | **<0.0001** |  |  |  |  |
|  |  | N97I | -12.32 | 0.18 | 5 | **<0.0001** |  |  |  |  |
|  |  | D131H | -10.29 | 0.21 | 6 | **<0.0001** |  |  |  |  |
| 13. Effect of LQTS-associated mutations on CaM affinity for Kv7.1-Helix B (Ca^2+^-bound)? | *K*_d_ increased for all variants | CaM-WT | 0.56 | 0.07 | 6 | - | nM | WT vs mutants | One-way ANOVA, Dunnett’s multiple comparisons tests | 10 |
|  |  |  | 538.0 | 72.9 | 6 | - |  |  |  |  |
|  |  | D95V | 6.90 | 1.50 | 5 | **<0.0001** |  |  |  |  |
|  |  |  | 725.0 | 117.0 | 5 | **0.0067** |  |  |  |  |
|  |  | N97I | 4.70 | 0.46 | 5 | **<0.0001** |  |  |  |  |
|  |  |  | 775.0 | 91.9 | 5 | **0.0009** |  |  |  |  |
|  |  | D131H | 9.70 | 1.20 | 6 | **<0.0001** |  |  |  |  |
|  |  |  | 908.0 | 70.0 | 6 | **<0.0001** |  |  |  |  |
| 14. Effect of LQTS-associated mutations on CaM:Kv7.1-Helix B thermodynamic parameters, ΔG (Ca^2+^-bound)? | ΔG increased for all variants | CaM-WT | -12.65 | 0.08 | 6 | - | kcal/mol | WT vs mutants | One-way ANOVA, Dunnett’s multiple comparisons tests | 10 |
|  |  |  | -8.56 | 0.08 | 6 | - |  |  |  |  |
|  |  | D95V | -11.14 | 0.17 | 5 | **<0.0001** |  |  |  |  |
|  |  |  | -8.39 | 0.11 | 5 | **0.0048** |  |  |  |  |
|  |  | N97I | -11.36 | 0.09 | 5 | **<0.0001** |  |  |  |  |
|  |  |  | -8.35 | 0.07 | 5 | **0.0007** |  |  |  |  |
|  |  | D131H | -10.95 | 0.08 | 6 | **<0.0001** |  |  |  |  |
|  |  |  | -8.25 | 0.04 | 6 | **<0.0001** |  |  |  |  |
| 15. Effect of LQTS-associated mutations on CaM:Kv7.1-Helix B thermodynamic parameters, ΔH (Ca^2+^-bound)? | ΔH altered for N97I variant.  ΔH partially reduced for D95V and D131H variants | CaM-WT | -7.70 | 0.45 | 6 | - | kcal/mol | WT vs mutants | One-way ANOVA, Dunnett’s multiple comparisons tests | 10 |
|  |  |  | -14.75 | 1.24 | 6 | - |  |  |  |  |
|  |  | D95V | -13.22 | 0.69 | 5 | **<0.0001** |  |  |  |  |
|  |  |  | -14.86 | 0.72 | 5 | 0.9906 |  |  |  |  |
|  |  | N97I | -12.92 | 0.37 | 5 | **<0.0001** |  |  |  |  |
|  |  |  | -11.50 | 0.26 | 5 | **<0.0001** |  |  |  |  |
|  |  | D131H | -14.32 | 0.25 | 6 | **<0.0001** |  |  |  |  |
|  |  |  | -15.53 | 0.24 | 6 | 0.2136 |  |  |  |  |
| 16. Effect of LQTS-associated mutations on CaM:Kv7.1-Helix B thermodynamic parameters, -TΔS (Ca^2+^-bound)? | -TΔS altered for N97I and D131H variants.  -TΔS partially increased for D95V variant | CaM-WT | -4.94 | 0.43 | 6 | - | kcal/mol | WT vs mutants | One-way ANOVA, Dunnett’s multiple comparisons tests | 10 |
|  |  |  | 6.37 | 0.87 | 6 | - |  |  |  |  |
|  |  | D95V | 2.07 | 0.76 | 5 | **<0.0001** |  |  |  |  |
|  |  |  | 6.49 | 0.75 | 5 | 0.9749 |  |  |  |  |
|  |  | N97I | 1.52 | 0.38 | 5 | **<0.0001** |  |  |  |  |
|  |  |  | 3.14 | 0.28 | 5 | **<0.0001** |  |  |  |  |
|  |  | D131H | 3.99 | 1.50 | 6 | **<0.0001** |  |  |  |  |
|  |  |  | 7.29 | 0.30 | 6 | **0.0464** |  |  |  |  |

*You may use multiple lines for the same question to indicate multiple comparisons

** Authors may wish to make the text bold where p is considered significant against a stated confidence limit.
